# Indolent T-Cell Lymphoproliferative Disease of the GI Tract: Insights for Better Diagnosis, Prognosis, and Appropriate Therapy

**DOI:** 10.3389/fonc.2020.01276

**Published:** 2020-08-07

**Authors:** Can Küçük, Li Wei, Hua You

**Affiliations:** ^1^Affiliated Cancer Hospital & Institute of Guangzhou Medical University, Guangzhou, China; ^2^Izmir Biomedicine and Genome Center (IBG), Izmir, Turkey; ^3^Department of Medical Biology, Dokuz Eylül University, İzmir, Turkey; ^4^Department of Pathology, City of Hope Medical Center, Duarte, CA, United States

**Keywords:** Indolent T cell lymphoproliferative disease of the GI tract, misdiagnosis, prognosis, targeted therapy, T cell lymphoma, NK cell enteropathy, inflammatory bowel disease, genetic aberrations

## Abstract

Indolent T-cell lymphoproliferative disease of the gastrointestinal tract (indolent GI T-LPD) is a benign neoplasm of CD4^+^ or CD8^+^ T cells that form primary tumors in the GI tract. Indolent GI T-LPD has recently been provisionally recognized as a distinct entity by the 2016 revision of the WHO classification of lymphoid neoplasms. Appropriate diagnosis of these cases is challenging as they may be misdiagnosed as T cell lymphoma that has an aggressive clinical course. Consequently, aggressive therapeutic approaches were usually chosen to treat these cases with no obvious benefit for most of the patients and potential side effects. Moreover, inflammatory diseases of the GI tract with similar symptoms may lead to misdiagnosis that leads to delays in administration of proper therapeutics against these cases. Therefore, it is of utmost importance to identify prognostic genetic biomarkers at the time of diagnosis for optimal medical care of these patients. TCR clonality analyses may not be useful for distinguishing these benign neoplasms from aggressive gastrointestinal T cell lymphomas; however, molecular genetic tests may prove useful as recurrent *STAT3-JAK2* fusions, which may have diagnostic, prognostic or therapeutic value, have recently been identified. However, there is still lack of comprehensive information on the genetic and epigenetic factors associated with pathogenesis of indolent GI T-LPD. In this mini-review, we focus on the so far reported literature on indolent GI T-LPD cases, and discuss future directions for better differential diagnosis, risk stratification, and therapeutic target discovery with a special focus on the genetic and epigenetic alterations.

## Background

Indolent T cell lymphoproliferative disease of the gastrointestinal tract (indolent GI T-LPD) is a benign lymphoid neoplasm provisionally recognized by the World Health Organization (WHO) classification of lymphoid neoplasms in 2016 as a distinct disease entity (1). These neoplasms mostly occur in the GI tract owing to clonal T cell proliferations. They may drive from CD8^+^ T cells and less often from CD4^+^ T cells (1). The infiltrating lymphocytes are mature small lymphocytes that are negative for CD56 expression; implying that they do not originate from NK cells. In general, these tumors show a non-invasive pattern and indolent clinical behavior. The proliferative index detected with Ki67 staining was very low (~5%) in indolent GI T-LPD cases (2). GI T-LPD is more common in males compared to females; and it is mainly observed in adults with a wide range of age (3).

Recognition of indolent GI T-LPD as a distinct neoplasm has important therapeutic implications especially for the type of therapeutic approach chosen after diagnosis. Importantly, misdiagnosis is currently one of the biggest challenge for these benign cases as they may be diagnosed as aggressive T cell lymphomas or inflammatory diseases, which may lead to improper management of these patients due to waste of time and wrong therapy applied (2). Our current knowledge on this neoplasm is still at the very beginning, and more research needs to be performed to have a deeper understanding of the genetic or epigenetic aberrations associated with the pathogenesis of indolent GI T-LPD cases.

In the following sections, we discuss (1) indolent GI T-LPD cases reported in the literature including unusual observations; (2) genetic alterations identified to overcome indolent GI T-LPD misdiagnosis problem; (3) prognosis and therapy of these cases; (4) future directions to shed light onto the possible research areas that may generate findings potentially useful in the clinical practice.

## Case Reports

Nagaishi et al. observed an indolent GI T-LPD case with persistent diarrhea and villous atrophy in small intestine as the endoscopic finding (4). This case was treated with mogamulizumab due to surface expression of CCR4, which achieved durable remission in symptoms for more than 2 years. Interestingly, indolent GI T-LPD cases may unusually express CD20 which may lead to misdiagnosis as B-cell lymphomas thereby leading to unnecessary aggressive therapy (5). Guo et al. has recently reported an interesting GI T-LPD case with co-occurrence of diffuse large B cell lymphoma (6). This patient is a 46-year-old Chinese male initially diagnosed as GI T-LPD based on histological examination and T-cell receptor-gamma (TCRγ) clonal gene rearrangement assessment. This benign neoplasm may develop as a result of therapy against inflammatory diseases. Edison et al. reported an indolent GI T-LPD case developed in a patient with resistant Crohn's colitis as a result of tumor necrosis factor alpha (TNFα) therapy (7). Indolent GI T-LPD cases may rarely be associated with EBV infections. Wang et al. has recently reported a very rare case of adult-onset, immunocompetent EBV^+^ GI T-LPD (8). This case was a middle-aged woman with chronic diarrhea and severe intestinal bleeding. Importantly, she died 7 months post-diagnosis, which suggests that some GI T-LPD cases may show clinically aggressive behavior. The poor prognosis of this EBV positive case may be related to EBV-encoded oncogenic miRNAs as elevated levels of circulating miR-BART2-5p was shown to be associated with disease progression for EBV^+^ natural killer/T cell lymphoma (9). Given the lack of response of this GI T-LPD case to a variety of therapeutics, high expression of PD-L1 could be responsible for this patient's poor prognosis as EBV-encoded LMP1 upregulated PD-L1, and elevated PD-L1 resulted in adverse clinical course in NKTCL cases (10). EBV-induced epigenomic aberrations were reported in EBV^+^ malignancies (11). If these epigenomic changes are also present in EBV^+^ GI T-LPD cases, it will be worthwhile to address whether they affect patient survival or not. Although these observations imply that EBV infection is very likely responsible for poor prognosis of EBV^+^ GI T-LPD cases, we can not totally exclude the role of other potential pathogenic events. The characteristics of important cases reported are shown in Table 1.

**Table 1:** Important case reports of indolent T-cell lymphoproliferative disease of the gastrointestinal tract.

**CD4/CD8** **positivity**	**Age**	**Gender**	**Involved sites**	**Clinical symptoms**	**Unique feature**	**References**
CD4^−^/CD8^−^	46	Male	Stomach, ileum, colon and bone marrow	✓ Abdominaldistension ✓ Dyspepsia	Aberrant CD20 expression	(5)
CD4^+^	69	Male	Small intestine and duodenum	✓ Diarrhea ✓ Intermittent fever ✓ Wasting	Better diagnosis with single-balloon enteroscopy	(4)
CD4^+^	37	Male	Colon	✓ Abdominal pain ✓ Diarrhea	✓ Initially showed diagnostic features of Crohn's disease ✓ Aggressive T-cell lymphoma development and fatality	(12)
CD8^+^	27	Female	Sigmoid colon, ascending colon, cecum	N.A.	TNF-α inhibitor treatment led to indolent GI T-LPD Development	(7)
CD8^+^	46	Male	Intestine	✓ Paraumbilical colic pain ✓ Bloating ✓ Occasional diarrhea	Co-existence of indolent GI T-LPD and DLBCL	(6)
CD4^+^/CD8^+^	43	Female	Intestine, ileum terminal, colon, rectum	✓ Fever with chill ✓ Abdominal pain ✓ Diarrhea ✓Hematochezia	✓ Adult onset EBV^+^ ✓ Extremely rare case with poor prognosis ✓ Mimic inflammatory bowel disease	(8)

*DLBCL, Diffuse large B cell lymphoma; N.A., Not available*.

## Discovered Genetic Alterations to Avoid Indolent GI T-LPD Misdiagnosis

Indolent GI T-LPD cases may be misdiagnosed as enteropathy associated T cell lymphoma (EATL) or monomorphic epitheliotropic intestinal T-cell lymphoma (MEITL), which are rare but aggressive lymphomas of the GI tract (2). The presenting symptoms of these aggressive gastrointestinal T cell lymphomas (e.g., abdominal pain, diarrhea) may be quite similar to those of indolent GI T-LPD cases (Table 2). Recent studies revealed recurrent activating mutations of *STAT5B* (18) and loss-of-function mutations of *SETD2* (19) in MEITL cases, which can be useful for distinguishing MEITL cases from indolent GI T-LPD cases. By screening activating mutations located in the SH2 (Src Homology 2) domain of *STAT3*, it may be possible differentiate indolent GI T-LPD cases from EATL cases as the former did not have *STAT3* mutations (2); whereas EATL cases harbor *STAT3* mutations (13). Importantly, this neoplasm needs to be also differentiated from NK cell enteropathy, which is a recently described NK cell neoplasm appearing in gastrointestinal sites (20). Given that 30% frequency of JAK3 K563_C565del mutations were detected in NK cell enteropathy cases, these mutations may potentially be used to differentiate a subset of NK cell enteropathy cases from indolent GI T-LPD cases (17).

**Table 2:** Symptoms and recurrent genetic alterations in indolent GI T-LPD and similar gastrointestinal diseases.

**Gastrointestinal disease type**	**Presenting symptoms**	**Recurrent genetic alterations**	**Reference for mutations**
Enteropathy associated T cell lymphoma	- Abdominal pain -Anorexia - Adenopathy - Fatique - Infection	Activating mutations of *JAK1* and *STAT3*	(13)
Inflammatory bowel disease (IBD) - Ulcerative collitis - Crohn's disease	- Abdominal pain and cramping - Blood in stool - Diarrhea - Fever and fatigue - Reduced appetite - Unintended weight loss	Germline mutations of *IL10RA, IL10RB*, and integrin genes	(14, 15)
Indolent T-cell lymphoproliferative disease of the GI tract	- Abdominal pain - Diarhea - Dyspepsia - Food intolerance - Vomiting	*STAT3-JAK2* fusions in CD4^+^ cases	(16)
NK cell enteropathy	- Constipution - Diarrhae - GI bleeding - Vague abdominal pain - Vomiting	*JAK3* K563_C565del	(17)
Monomorphic epitheliotropic intestinal T-cell lymphoma^*^	- Abdominal pain - Bowel obstruction - Diarhea - Intestinal bleeding - Perforation - Weight loss	*STAT5B* N642H, and loss-of function mutations of *SETD2*	(18, 19)

N.D., Not determined.

* *Formerly known as type II EATL*.

The symptoms of inflammatory bowel disease (IBD) can be highly similar to those of indolent GI T-LPD cases, which in turn may lead to misdiagnosis of the latter ones (Table 2). Inflammatory bowel disease (IBD) is a heterogeneous disease that can further be subclassified as ulcerative collitis and Crohn's disease (21). There are few reports available investigating the genetic aberrations responsible for pathogenesis of IBD. Glocker et al. performed a pedigree-based analyses, and identified three distinct homozygous mutations in *IL10RA* and *IL10RB* genes, which form the IL10 receptor complex (14). These germline mutations prevented IL10 receptor signaling; thereby, disrupting a negative feedback regulation in peripheral blood mononuclear cells. Furthermore, a genome-wide association study (GWAS) revealed several new susceptibility loci, three of which include integrin genes (15). Given the difficulty of obtaining sufficiently large biopsy material from gastrointestinal sites, genetic screen for these inherited mutations in peripheral blood cells may be useful in routine clinical practice for distinguishing IBD and indolent GI T-LPD cases.

There are very few studies available, which focused on genetic aberrations that are associated with pathogenesis of indolent GI T-LPD cases. It is of utmost significance to identify genetic aberrations in this benign neoplasm to better diagnose, predict prognosis, and identify therapeutic targets. To address whether activating mutations of *STAT3* previously reported in lymphomas derived from gamma delta T or NK cells (18) are present also in indolent GI T-LPD cases, Perry et al. screened six mutational hotspots in the SH2 domain of *STAT3* in five indolent GI T-LPD case, which did not reveal any activating *STAT3* mutation (2). Using complementary methodological approaches (i.e. FISH, RNA-Seq, Sanger sequencing), Sharma et al. has recently identified *STAT3-JAK2* fusion genes in four of five CD4^+^ GI T-LPD cases but not in CD8^+^ or CD4^+^/CD8^+^ cases evaluated (16). If this finding is supported by future studies, *STAT3-JAK2* fusion genes may be an important diagnostic biomarker which can be used in genetic tests for routine clinical applications. The indolent GI T-LPD cases with *STAT3-JAK2* fusion genes did not show STAT3 activation but showed STAT5 activity based on immunohistochemistry. STAT3 Y705 residue phosphorylation is an indicator of STAT3 activation (18). It was unusual not to observe STAT3 activation in the CD4^+^ GI T-LPD cases with the STAT3-JAK2 fusion protein as this fusion gene retains the Y705 residue. Importantly, a recent study by Hu et al. ectopically expressed STAT3-JAK2 in different cell types, and showed that STAT3 is marginally phosphorylated, and have transcriptional activity albeit to a lesser extent than that of STAT5. Of note, STAT3-JAK2 fusion protein was observed to bind to and phosphorylate STAT5 much efficiently, consistent with the observation that STAT5 target genes were enriched in STAT3-JAK2 transduced T cell breast lymphoma 3 (TLBR3) cells but not STAT3 (22). The observed discrepancy related to STAT3 activation status may be due to relatively lower sensitivity of immunohistochemistry compared to that of western blot, high level of expression of phosphatases that dephosphorylate STAT3, or the intracellular amount of available STAT3 protein in indolent GI T-LPD cells. From clinical point of view, genetic detection of the STAT3-JAK2 using FISH coupled with STAT5 IHC may be the most suitable option during differential diagnosis, and decision for appropriate therapy for CD4^+^ T-LPDs. The clinical symptoms and recurrent genetic alterations in indolent GI T-LPD and similar gastrointestinal diseases are shown in Table 2.

## Prognosis and Therapy

GI T-LPD usually presents as an indolent neoplasm with no progression to aggressive T cell lymphomas. These cases are often misdiagnosed as T cell lymphoma with little or no response to chemotherapy (23). As an example, an indolent GI T-LPD case with CD4^−^/CD8^+^ phenotype was initially misdiagnosed as MEITL (24). This case showed active but stable disease for 5 years post-administration of different chemotherapeutics. The fact that gastro-intestinal tract is the most common primary tumor site for extra-nodal lymphomas further underscores the importance of correct diagnosis of these benign neoplasms (3). Importantly, rare cases of indolent GI T-LPD that convert to aggressive lymphomas have been reported in the literature. Perry et al. has recently reported a 37 year-old male of an indolent GI T-LPD case who transitioned to peripheral T cell lymphoma 3 years after initial diagnosis (12). In general, genetic or epigenetic prognostic biomarkers that predict transition to peripheral T cell lymphoma in indolent GI T-LPD cases are unknown. It is possible that STAT3-JAK2 fusion gene promotes transition to aggressive T cell lymphoma as one of 5 CD4^+^ GI T-LPD case with the fusion protein was reported to develop T-cell lymphoma (16). However, more comprehensive studies need to be performed to address whether STAT3-JAK2 can be used as a prognostic biomarker for indolent GI T-LPD cases.

Radiotherapy may be a more effective option for treatment of indolent GI-T-LPD cases compared with chemotherapy. Recently, an indolent GI T-LPD case that have tumors localized in stomach has been treated successfully using involved field radiotherapy (IFRT) (25). This observation suggests that radiotherapy may be a good choice for treatment of cases with localized disease presenting in gastric sites. Nevertheless, it should be noted that longer follow-up is needed for this radiotherapy-treated case as it was followed-up only for 1 year post-treatment.

Edison et al. reported an unusual case of indolent GI T-LPD with resistant Chron's disease, which developed after tumor necrosis factor-α (TNF-α) inhibitor therapy (7). Interestingly, stopping TNF-α inhibitor therapy led to regression of the tumor, which suggests a causal relationship between this therapy and GI T-LPD. Furthermore, this observation suggests the need for avoiding TNF-α inhibitor treatment of patients with resistant Chron's disease to prevent development of indolent GI T-LPD.

Characterization of genetic lesions observed in indolent GI T-LPD as oncogenic drivers have potential to reveal novel therapeutic targets. Hu et al.'s study provided invaluable information on potential oncogenic role of STAT3-JAK2 in GI T-LPD pathogenesis. In that study, they showed that ectopic STAT3-JAK2 promotes growth of CD4^+^ T cells, and results in cytokine independent growth of Ba/F3, an IL3-dependent pro-B cell line cell. Of note, the main oncogenic function of this fusion protein was through activation of STAT5. Using an *in vivo* xenograft model, authors showed that STAT3-JAK2 promotes tumor growth, and this growth can be inhibited using JAK inhibitors. Altogether these observations imply that STAT3-JAK2 fusion protein is not important only as a diagnostic biomarker but also as a potential therapeutic target in CD4^+^ indolent GI T-LPD cases having this genetic lesion.

## Future Directions

**Figure 1:**
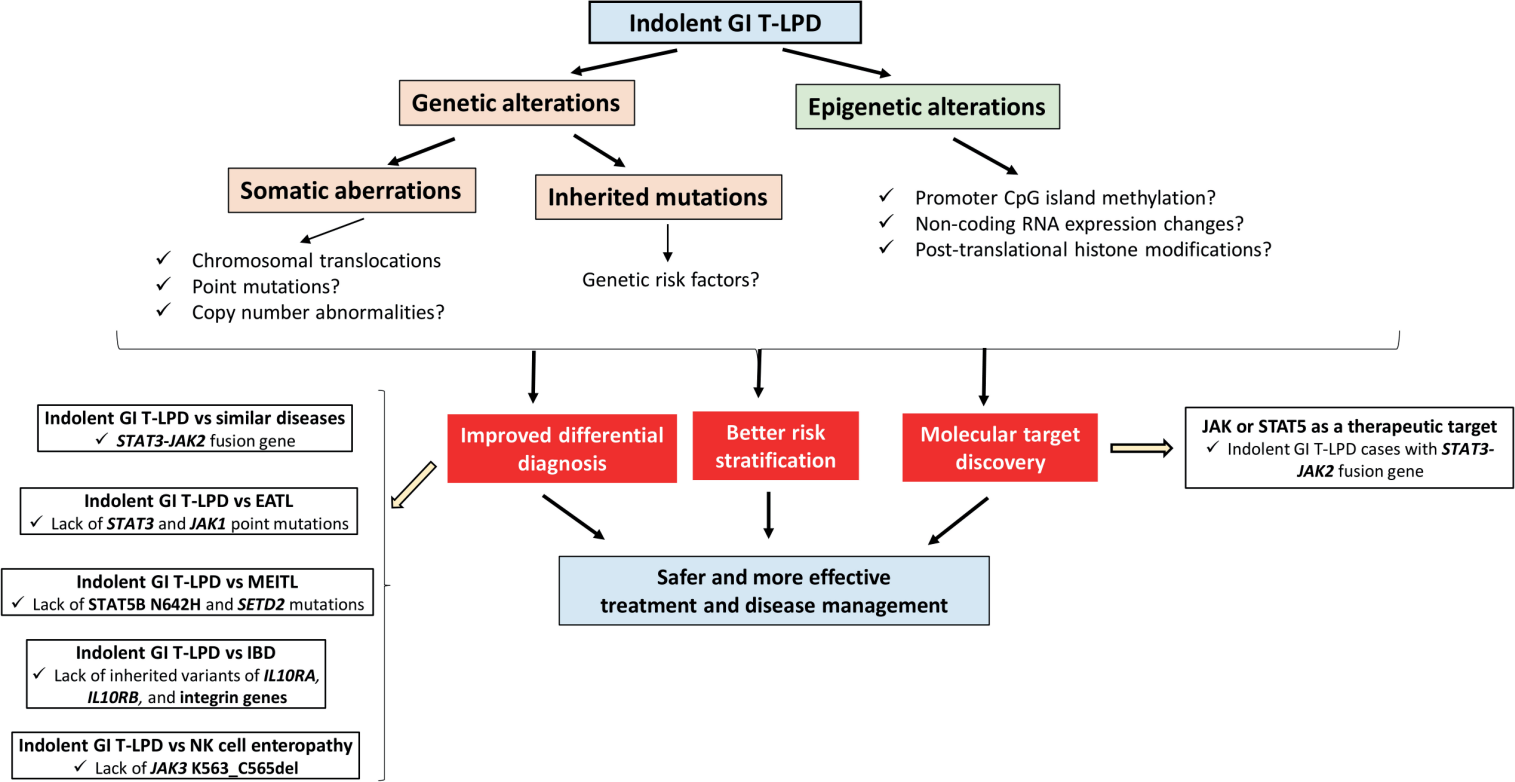
The schematic diagram showing the available and potential future genetic or epigenetic aberrations to be identified for better diagnosis, prognosis, and therapy of indolent GI T-LPD cases.

Our current knowledge on genetic and epigenetic alterations associated with indolent GI T-LPD is at the very beginning. There are several research areas to be focused on to improve differential diagnosis, predict prognosis, or identify therapeutic targets for better management of these cases (Figure 1). Most importantly, whole exome or genome sequencing of indolent GI T-LPD tumors have not been performed yet in the presence of patient-matched samples so the somatic mutation profiles of these tumors are still unknown. Obtaining sufficient tissue material from gastrointestinal sites of indolent T-LPD cases may be challenging for genetic testing. Hence, it would be interesting to see whether cancer-associated somatic mutations may be detected by targeted ultra-deep sequencing of plasma cell-free circulating DNA fragments, a method that proved highly useful for lymphoma types such as DLBCL (26) and classical Hodgkin's lymphoma (27). It would also be interesting to investigate whether there is genetic predisposition to indolent GI T-LPD by performing a genome-wide association study (GWAS) on a large cohort of patients to identify SNPs associated with indolent GI T-LPD, or perform pedigree-based genetic analyses to identify inherited mutations that contributed to development of these neoplasms. Identification of genetic predisposition factors that distinguish indolent T-LPD form other similar diseases may offer unique opportunities for improved diagnosis or prognosis through targeted genetic testing with a next-generation device or PCR-Sanger in routine clinic (28).

Epigenetic alterations such as promoter methylation-mediated tumor suppressor gene silencing (29), aberrant histone methylations (30), miRNA expression changes (31) can be used as biomarkers and/or therapeutic targets to improve diagnosis, risk stratification, and therapeutic efficacy of indolent GI T-LPD cases. To the best of our knowledge, no study has been reported investigating the epigenetic characteristics of indolent GI T-LPD cases. Given that epigenetic aberrations play key roles in regulating different “hallmarks” of cancer, it will be interesting to study the role of major epigenetic events in development of this neoplasm.

## Conclusions

To sum up, there are many unknowns regarding the genetic and epigenetic aberrations associated with the development and pathogenesis of indolent GI T-LPD. Future investigations will reveal the genomic and epigenomic landscape that potentially can distinguish this neoplasm from other similar diseases to achieve accurate diagnosis; thereby leading to more effective therapeutic approaches with better disease management. Importantly, the presence of these disease-related aberrations will support distinguished recognition of indolent GI T-LPD as a distinct disease category by the medical authorities.

## Author Contributions

CK, LW, and HY critically evaluated the literature related to the topic, identified references, and wrote the manuscript. HY financially supported the study. All authors contributed to the article and approved the submitted version.

## Conflict of Interest

The authors declare that the research was conducted in the absence of any commercial or financial relationships that could be construed as a potential conflict of interest.

## Abbreviations

GI T-LPD: T-cell lymphoproliferative disease of the gastrointestinal tract
WHO: World Health Organization
TCRγ: T-cell receptor-gamma
TNFα: tumor necrosis factor alpha
EATL: enteropathy associated T cell lymphoma
MEITL: monomorphic epitheliotropic intestinal T-cell lymphoma
SH2: Src Homology 2
IBD: inflammatory bowel disease
GWAS: genome-wide association study.
